# Cyclic Electron Transport via the NDH Complex Sustains Photosynthesis and Productivity Under Fluctuating and Sub‐Optimal Environments

**DOI:** 10.1111/ppl.71004

**Published:** 2026-07-06

**Authors:** Hiromasa Kodama, Wataru Yamori

**Affiliations:** ^1^ Graduate School of Agricultural and Life Sciences The University of Tokyo Tokyo Japan

**Keywords:** cyclic electron transport, electron transport, NDH complex, photoinhibition, photosynthesis

## Abstract

The chloroplast NADH dehydrogenase‐like (NDH) complex mediates cyclic electron transport (CET) around photosystem I (PSI) and contributes to photosynthetic regulation and photoprotection under various environmental stresses. Although NDH function has been extensively characterized under controlled conditions, NDH‐deficient mutants often show only subtle phenotypes in such environments, leaving its physiological importance to photosynthesis in plants grown under naturally fluctuating field conditions poorly understood. Here, we evaluated growth, yield, and photosynthetic performance of NDH‐deficient rice cultivated in outdoor fields. Mutant plants exhibited reduced biomass accumulation and grain yield compared with wild type (WT). Detailed physiological analyses in field‐grown plants revealed that NDH deficiency markedly decreased PSI electron transport and CO_2_ assimilation, particularly under low temperature and sub‐saturating irradiance. At moderate and high temperatures, reductions in CO_2_ assimilation were largely confined to low‐light conditions, whereas at low temperatures, impairment extended across nearly the entire light response range. Under repetitive fluctuating light regimes, NDH‐deficient plants showed progressive declines in CO_2_ assimilation accompanied by a decrease in PSI and PSII photochemical capacity, indicating photoinhibition of both PSI and PSII. These findings demonstrate that NDH‐dependent CET plays a crucial role in sustaining crop productivity in field environments by stabilizing photosynthetic efficiency under sub‐optimal conditions through maintaining PSI redox balance.

## 1 Introduction

In natural environments, plants are continuously exposed to dynamic fluctuations in light intensity, spectral quality, and temperature that occur across timescales ranging from seconds to seasons. Such environmental variability strongly influences photosynthetic performance and ultimately determines plant productivity and crop yield (Pearcy 1990; Long et al. 2006; Yamori 2016). In particular, rapid increases in irradiance caused by sunflecks or canopy gaps can induce transient over‐reduction of the photosynthetic electron transport chain, especially on the acceptor side of photosystem I (PSI), resulting in the generation of reactive oxygen species (ROS) and PSI‐specific photoinhibition (Yamori, Makino, and Shikanai 2016; Kono et al. 2017; Miyake 2020). Conversely, periods of low light or suboptimal temperature can limit ATP supply and carbon assimilation capacity, leading to imbalances between energy absorption and utilization (Yamori et al. 2011; Yamori, Makino, and Shikanai 2016). Therefore, plants must maintain a delicate balance between maximizing photosynthetic efficiency and preventing photodamage under naturally fluctuating environmental conditions (Tikkanen and Grebe 2018).

A central regulatory mechanism enabling such acclimation is cyclic electron transport (CET) around PSI. CET redirects electrons from ferredoxin (Fd) back to the plastoquinone (PQ) pool via the cytochrome *b*_6_/*f* complex, thereby generating proton motive force (*pmf*) and promoting ATP synthesis without net NADPH production (Yamori and Shikanai 2016; Shikanai et al. 2025). The proton gradient (ΔpH) generated across the thylakoid membrane contributes not only to *pmf* formation but also to regulatory processes that downregulate electron transport. In particular, lumen acidification rapidly induces qE, a major component of non‐photochemical quenching (NPQ; Briantais et al. 1979), and suppresses electron transfer at the cytochrome *b_6_/f* complex through a mechanism known as photosynthetic control (West and Wiskich 1968). CET‐dependent ΔpH formation is therefore closely associated with both photoprotective energy dissipation and regulation of PSI redox balance (Munekage et al. 2004; Zhou et al. 2022). Through these mechanisms, CET plays a critical role in sustaining photosynthesis and plant growth under various environmental constraints.

In angiosperms, CET is mediated by at least two distinct pathways: one dependent on the PROTON GRADIENT REGULATION5 (PGR5)/PGR5‐LIKE1 (PGRL1) protein complex and another involving the chloroplast NADH dehydrogenase‐like (NDH) complex (Shikanai et al. 1998; Munekage et al. 2004; DalCorso et al. 2008). The PGR5‐dependent pathway has been extensively studied and is known to be essential for photoprotection under high light and fluctuating light conditions, as evidenced by the severe growth defects of *pgr5* mutants in *Arabidopsis thaliana* exposed to dynamic irradiance regimes (Tikkanen et al. 2010; Suorsa et al. 2012). However, recent studies have questioned the classical view that the PGR5‐dependent pathway primarily functions to increase ATP synthesis via CET. Instead, PGR5‐dependent regulation appears to contribute more directly to ΔpH formation, induction of NPQ, and photosynthetic control (Degen et al. 2025). In contrast, the physiological significance of NDH‐dependent CET has long remained less clear, partly because NDH‐deficient mutants often show only subtle phenotypes under steady laboratory conditions (Peng and Shikanai 2011; Wang et al. 2015). Moreover, the distinct physiological roles of the different CET pathways remain incompletely understood. Therefore, clarifying the contribution of NDH‐dependent CET to photosynthetic regulation under environmentally relevant conditions remains an important challenge.

Recent studies have begun to clarify that the NDH complex contributes to photosynthetic regulation in specific environmental contexts. The NDH complex accepts electrons from reduced Fd and transfers them to PQ while simultaneously translocating protons across the thylakoid membrane, thereby enhancing the formation of ΔpH and modulating *pmf* partitioning (Yamori et al. 2015; Yamori, Makino, and Shikanai 2016; Basso et al. 2020). This proton‐pumping activity supports photosynthetic control by regulating electron transport at the cytochrome *b*_6_/*f* complex, particularly during photosynthetic induction and under low‐light conditions (Yamori et al. 2015; Basso et al. 2020; Zhou et al. 2023). Moreover, NDH has been shown to functionally interact with ion transporters such as KEA3, highlighting its role in fine‐tuning the balance between photoprotection and photosynthetic efficiency (Armbruster et al. 2014; Basso et al. 2020).

Importantly, NDH‐dependent CET plays an important role in protecting PSI under abiotic stress conditions in angiosperms. In cyanobacteria, mosses, and some gymnosperms, flavodiiron proteins (FLVs) function as alternative electron sinks that alleviate excessive electron accumulation at PSI (Rantala et al. 2020). In contrast, angiosperms lack FLVs and therefore rely more heavily on CET pathways to maintain PSI oxidation under fluctuating and stressful environmental conditions. Under stress conditions, over‐reduction of the PSI acceptor side promotes reactive oxygen species (ROS) generation and PSI photoinhibition (Huang et al. 2019). In particular, fluctuating light can induce transient over‐reduction of PSI during rapid transitions from low to high irradiance (Sonoike 1995). NDH‐dependent CET contributes to alleviating this over‐reduction by both restricting electron flow toward PSI through ΔpH‐dependent photosynthetic control and transferring electrons from reduced Fd back to the PQ pool (Yamori, Makino, and Shikanai 2016; Yamori, Kondo, et al. 2016; Kono et al. 2017). The formation of ΔpH is supported by proton translocation associated with the cytochrome *b*_6_/*f* Q cycle as well as by proton‐pumping activity of the NDH complex itself. Indeed, the NDH complex functions as a proton pump and contributes directly to *pmf* formation (Strand et al. 2017). Consistent with these functions, NDH‐dependent CET has been implicated in plant responses to diverse abiotic stresses, including drought, heat, and high irradiance (Horváth et al. 2000; Rumeau et al. 2007; Zhang and Sharkey 2009).

Although simplified fluctuating‐light systems have provided important mechanistic insights into photosynthetic regulation, such experimental designs do not fully reproduce the environmental complexity experienced by plants under natural conditions (Lawson et al. 2012; Vialet‐Chabrand et al. 2017). Indeed, plants grown under light regimes that mimic natural fluctuations exhibit distinct photosynthetic acclimation patterns compared with those grown under constant or monotonous fluctuating light conditions (Burgess et al. 2023). Therefore, combining growth analyses under naturally fluctuating cultivation conditions with controlled physiological measurements is important for evaluating the ecological relevance of photosynthetic regulatory mechanisms. Despite the growing recognition that realistic environmental variability must be considered to understand photosynthetic regulation in crops, the functional significance of NDH‐dependent CET under actual field conditions remains largely unexplored.

To address this knowledge gap, we investigated the physiological role of the NDH complex in rice ( *Oryza sativa* L.) cultivated under natural field environments. By combining growth analysis, yield evaluation, and detailed measurements of photosynthetic responses across a range of temperatures and light intensities, we aimed to determine how NDH‐dependent CET contributes to maintaining PSI function and carbon assimilation under realistic environmental stresses. Our results demonstrate that NDH plays a critical role in sustaining photosynthetic efficiency, preventing PSI photoinhibition, and supporting crop productivity under naturally variable conditions. These findings provide evidence supporting the physiological importance of NDH‐dependent CET under field‐relevant environmental conditions and suggest that this pathway contributes substantially to maintaining photosynthetic stability in dynamically changing agricultural environments.

## 2 Materials & Methods

### 2.1 Plant Materials and Growth Conditions

The rice mutant defective in the OsCRR6 gene (Os08g0167500) due to the insertion of the Tos17 retrotransposon (*crr6; −/−*), its wild type ( *Oryza sativa* ssp. *japonica* cv. Hitomebore), and control progeny lacking the Tos17 insertion in *OsCRR6* but sharing the same genetic background as the homozygous mutant lines (control*; +/+*) were used in this study (Yamori et al. 2015; Yamori, Makino, and Shikanai 2016).

**FIGURE 1:**
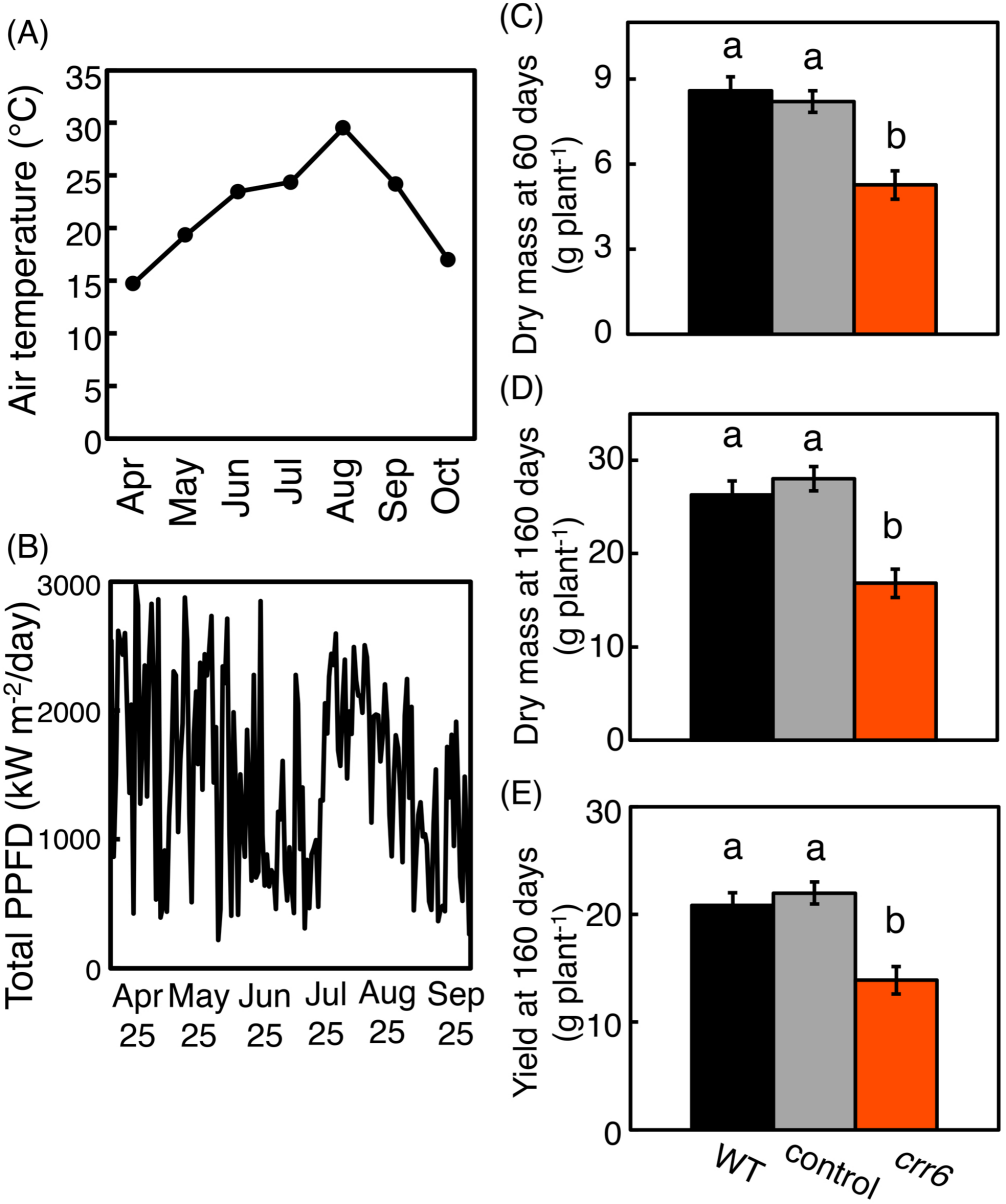
Growth and yield responses of *crr6* plants under outdoor cultivation. Seedlings were cultivated from 26 April to 4 October in a greenhouse under natural environmental conditions. (A, B) Monthly mean air temperature and daily cumulative photosynthetic photon flux density (PPFD) during the cultivation period. (C, D) Shoot and root dry mass measured at 60 days (C) and 160 days (D) after transplanting. (E) Grain yield at 160 days. Values represent means ± SE (*n* = 3–5). Different letters indicate significant differences among WT, control, and *crr6* plants according to Tukey–Kramer multiple comparison tests (*p* < 0.05).

Plants were grown under field conditions at the Institute for Sustainable Agro‐ecosystem Services, Graduate School of Agricultural and Life Sciences, The University of Tokyo (Tokyo, Japan) from late April to early October 2020. Environmental conditions during the experimental period are shown in Figure 1A,B.

Plants were sampled at 60 and 160 days after germination. Leaf shoots and roots were oven‐dried at 80°C, and weighed by precision balance. At 160 days, plants were harvested, and grain weight was measured to determine yield. After harvest, plants were air‐dried, then sampled and weighed.

### 2.2 Photosynthesis Analysis

Gas exchange, chlorophyll fluorescence, and P700 redox state were measured simultaneously using a GFS‐3000 gas‐exchange system and a Dual‐PAM‐100 measuring system (Walz) in the uppermost fully expanded leaves of 60–80‐day‐old plants apart from plants used for measuring dry mass at 60 days (Yamori, Makino, and Shikanai 2016; Qu et al. 2025). After 30 min dark adaptation, a saturating pulse (6000 μmol m^−2^ s^−1^, 300 ms) was applied to determine maximum fluorescence and the maximal P700 signal. Photosynthetic parameters were recorded every 20 s at a CO_2_ concentration of 400 μmol mol^−1^ under either constant high light or fluctuating light conditions. The quantum yield of PSI (Y(I)) was calculated as Y(I) = 1 − Y(ND) − Y(NA), where Y(ND) corresponds to the fraction of P700 that is already oxidized by actinic light and Y(NA) corresponds to the fraction of P700 that is closed owing to acceptor side limitation (Klughammer and Schreiber 1994). We calculated the quantum yield of photosystem II [Y(II) = (*F*_m_′–*F*′)/*F*_m_′], photochemical quenching [qP = (*F*_m_′–*F*′)/(*F*_m_′–*F*_o_')], non‐photochemical quenching [NPQ = (*F*_m_–*F*_m_′)/*F*_m_′] and the fraction of PSII centers in the open state (with PQ oxidized) [qL = qP × (*F*_o_'/*F*′); Genty et al. 1989; Hendrickson et al. 2004; Kramer et al. 2004). The electron transport rate (ETR) was calculated as ETR I (or ETR II) = 0.5 × abs I × Y(I) (or Y(II)), where 0.5 is the fraction of absorbed light reaching PSI or PSII and abs I is absorbed irradiance taken as 0.84 of incident irradiance.

### 2.3 Determination of Leaf Biochemical and Protein Contents

Immediately after gas‐exchange measurements, leaf samples were collected, frozen in liquid nitrogen, and stored at −80°C. Frozen samples were ground in liquid nitrogen and homogenized in extraction buffer. Leaf nitrogen, chlorophyll, and Rubisco contents were quantified as described by Yamori et al. (2011). Proteins were separated by SDS‐PAGE and transferred to polyvinylidene difluoride membranes. The abundance of CRR6, cytochrome *f* (cytochrome *b*_*6*_/*f* complex), and NdhK (a subunit of NDH subcomplex A) was determined using specific antibodies as described in Peng et al. (2009). A dilution series of wild‐type protein extracts was included to estimate relative protein abundance in mutant samples.

Rubisco large subunit content was determined spectrophotometrically following formamide extraction of Coomassie Brilliant Blue R‐250‐stained bands corresponding to Rubisco subunits. Chlorophyll was extracted in 80% (v/v) acetone and quantified according to Porra et al. (1989). Leaf nitrogen content was measured using a CHNO/S elemental analyzer (Vario EL III, Elementar).

### 2.4 Blue‐Native PAGE (BN‐PAGE) Analysis

BN‐PAGE was performed as described by Peng et al. (2009) with minor modifications. Freshly isolated thylakoid membranes were gently washed twice with buffer containing 25 mM BisTris‐HCl (pH 7.0) and 20% glycerol, and then solubilized in the same buffer supplemented with 1.0%, 1.5%, and 2.0% (w/v) n‐dodecyl‐β‐D‐maltoside (DDM) at a final chlorophyll concentration of 1 mg ml^−1^. After incubation on ice for 10 min, samples were centrifuged at 12000 × g for 10 min. The supernatants were supplemented with one‐tenth volume of BN sample buffer (100 mM BisTris‐HCl pH 7.0, 5% Serva Blue G, 0.5 M 6‐amino‐n‐caproic acid, and 30% sucrose). Equal amounts of chlorophyll were loaded per lane. Following electrophoresis, thylakoid protein complexes were visualized by staining with Coomassie Brilliant Blue (CBB).

### 2.5 Analysis of Photoinhibition

Leaves were placed in a temperature‐controlled chamber at 400 μmol mol^−1^ CO_2_ and 65% relative humidity within the Dual‐PAM‐100 and GFS‐3000 systems. Leaves were exposed to fluctuating light consisting of alternating high light (1500 μmol m^−2^ s^−1^) and low light (200 μmol m^−2^ s^−1^) at cycle lengths of 10, 5, or 2 min for 4.5 h (10‐min HL/LL cycles, 5‐min HL/LL cycles, 2‐min HL/LL cycles). The maximal P700 signal (Pm) and maximum PSII quantum yield (Fv/Fm) after 30 min dark incubation were measured before and after treatment. Photoinhibition was evaluated as the reduction in Pm and Fv/Fm following fluctuating light exposure.

### 2.6 Statistical Analyses

Statistical analyses were conducted using R software (version 4.1.2). One‐way ANOVA was performed to compare differences between WT and transgenic plants, followed by Tukey's post hoc test to identify significant pairwise differences. Statistical significance was set at *p* < 0.05 for all analyses.

## 3 Results

### 3.1 Loss of NDH Activity in the *crr6* Mutant

The chloroplast NDH complex consists of five subcomplexes (SubA, SubB, SubE, SubM, and SubL). CRR6 is required for the assembly of NdhI in subcomplex A (Shikanai et al. 2025). Immunoblot analysis confirmed that the CRR6 protein was absent in the *crr6* mutant (Figure S1A). To examine the effect of CRR6 deficiency on NDH accumulation, we analyzed the level of NdhK, a subunit of subcomplex A. NdhK was not detected in the *crr6* mutant, consistent with previous observations (Yamori et al. 2015).

In angiosperms, the NDH complex forms a supercomplex with two PSI complexes, which can be detected as a high‐molecular‐weight band by BN‐PAGE. This PSI–NDH supercomplex was observed in WT and control plants but was absent in the *crr6* mutant (Figure S1B).

NDH activity was further assessed by monitoring the transient post‐illumination increase in chlorophyll fluorescence, which reflects NDH‐dependent reduction of the PQ pool in darkness (Shikanai et al. 1998). This fluorescence rise was detected in WT and control plants but was completely absent in the *crr6* mutant (Figure S1C), demonstrating a complete loss of NDH activity.

### 3.2 NDH‐Dependent CET Contributes to Plant Growth and Yield Under Field Conditions

Plants were cultivated in the field from April 26 to October 4 under naturally fluctuating light and temperature conditions (Figure 1A,B). The *crr6* mutant showed significantly reduced dry mass at both 60 and 160 days after germination compared with WT and control plants (Figure 1C,D). Grain yield was also significantly lower in the *crr6* mutant (Figure 1E).

### 3.3 Leaf Biochemical Properties Are Unaffected by NDH Deficiency

Leaf nitrogen content, Rubisco content, cytochrome *f* abundance, and chlorophyll content per unit leaf area were similar among WT, control plants, and the *crr6* mutant (Table 1). The chlorophyll *a*/*b* ratio was also comparable between genotypes (Table 1), indicating that NDH deficiency did not alter major biochemical leaf traits.

**TABLE 1:** Effect of the *crr6* defect on physiological components of photosynthesis.

	Total *N*	Rubisco	Cyt *f*	Chl	Chl a/b
	(mmol m^−2^)	(μmol m^−2^)	(%)	(mmol m^−2^)	
WT	95.2 ± 4.9	5.27 ± 0.27	100.0 ± 3.3	0.47 ± 0.01	3.05 ± 0.06
control	96.6 ± 2.8	5.22 ± 0.19	105.8 ± 7.0	0.48 ± 0.01	3.08 ± 0.03
*crr6*	98.6 ± 5.8	5.63 ± 0.23	96.5 ± 4.8	0.49 ± 0.03	3.15 ± 0.07

*Note:* Contents of total nitrogen (Total N), Rubisco, Cytochrome *f* (Cyt *f*), Chlorophyll (Chl), and the ratio of Chlorophyll *a* and *b* (Chl *a*/*b*) were quantified. The Cyt *f* content is shown in a percentage relative to WT plants. Data represent means ± SE, *n* = 3–5. As a result of Tukey–Kramer multiple comparison tests (*p* < 0.05), there was no significant difference between WT, control plants, and *crr6* mutant. No significant differences were detected for any of the five measured variables.

### 3.4 NDH‐Dependent CET Supports PSI Activity and CO_2_ Assimilation Under Low Light and Low Temperature

**FIGURE 2:**
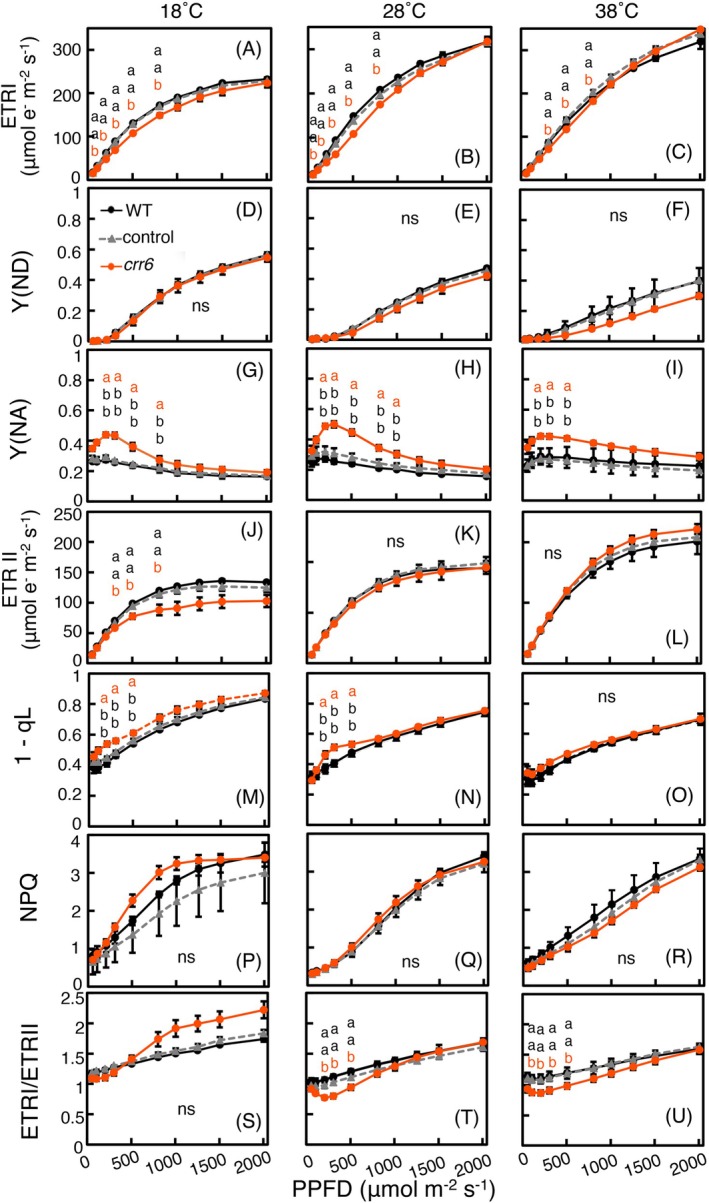
Effects of NDH deficiency on PSI redox state and PSII fluorescence parameters across light intensities at different temperatures. Light‐response curves of PSI redox parameters were determined at 18°C, 28°C, and 38°C in field‐grown plants. (A–C) Electron transport rate through PSI [ETR(I)]. (D–F) Donor‐side limitation of PSI [Y(ND)]. (G–I) Acceptor‐side limitation of PSI [Y(NA)]. (J–L) Electron transport rate through PSII [ETR(II)]. (M–O) Fraction of closed PSII centers (1 − qL). (P–R) Non‐photochemical quenching (NPQ). (S–U) Ratio of ETR(I) to ETR(II). Values represent means ± SE (*n* = 3–5). Significant differences among genotypes were evaluated using Tukey–Kramer tests (*p* < 0.05). Different letters indicate significant differences among WT, control, and *crr6* plants according to Tukey–Kramer multiple comparison tests (*p* < 0.05). Black symbols represent WT and control plants, while orange symbols represent *crr6* mutant.

**FIGURE 3:**
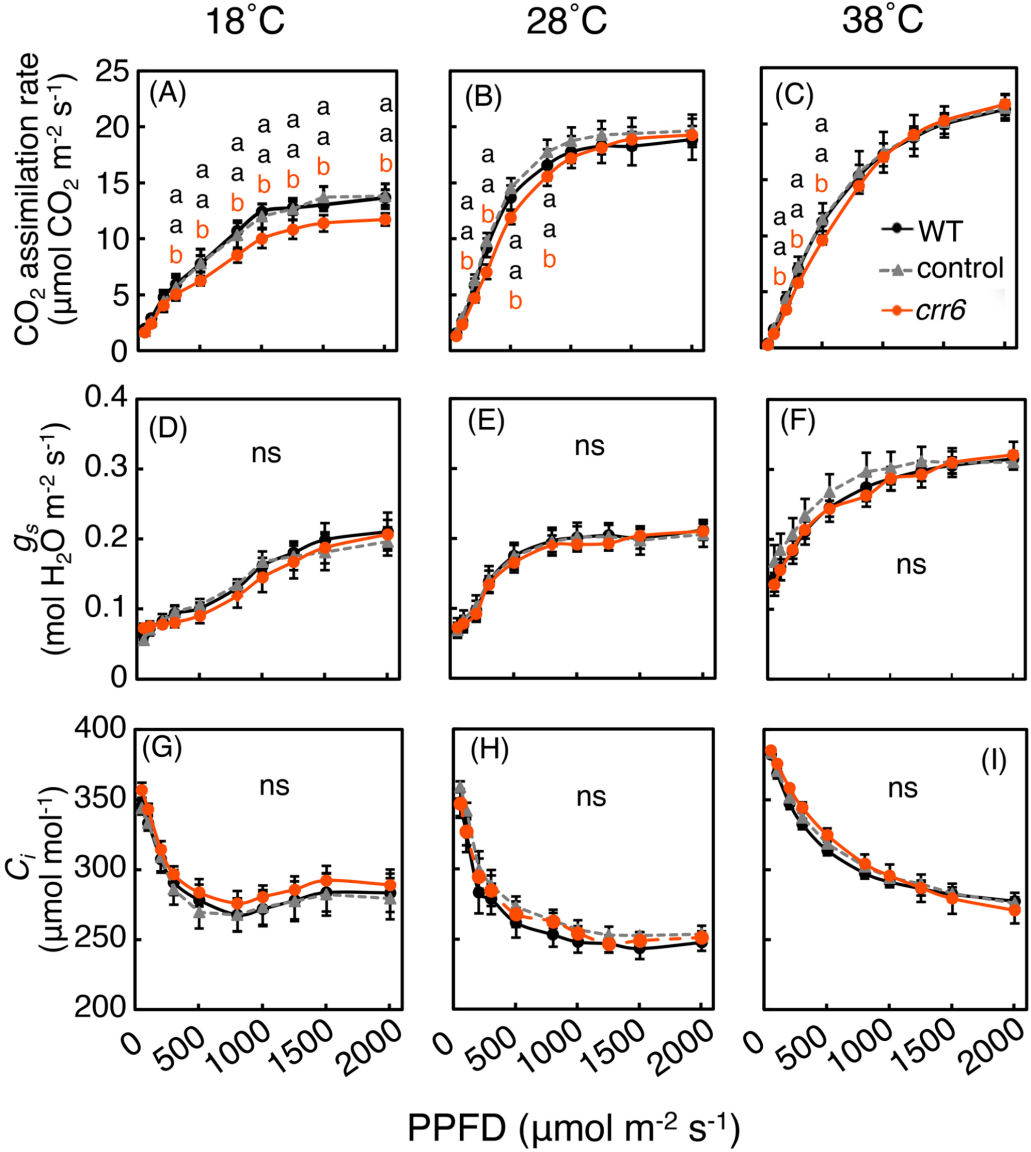
Effects of NDH deficiency on gas‐exchange responses across light intensities at different temperatures. Light‐response curves of gas‐exchange parameters were determined at 18°C, 28°C, and 38°C in field‐grown plants. (A–C) Net CO_2_ assimilation rate. (D–F) Stomatal conductance (g_s_). (G–I) Intercellular CO_2_ concentration (C_i_). Values represent means ± SE (*n* = 3–5). Significant differences among genotypes were evaluated using Tukey–Kramer tests (*p* < 0.05). Different letters indicate significant differences among WT, control, and *crr6* plants according to Tukey–Kramer multiple comparison tests (*p* < 0.05). Black symbols represent WT and control plants, while orange symbols represent *crr6* mutant.

**FIGURE 4:**
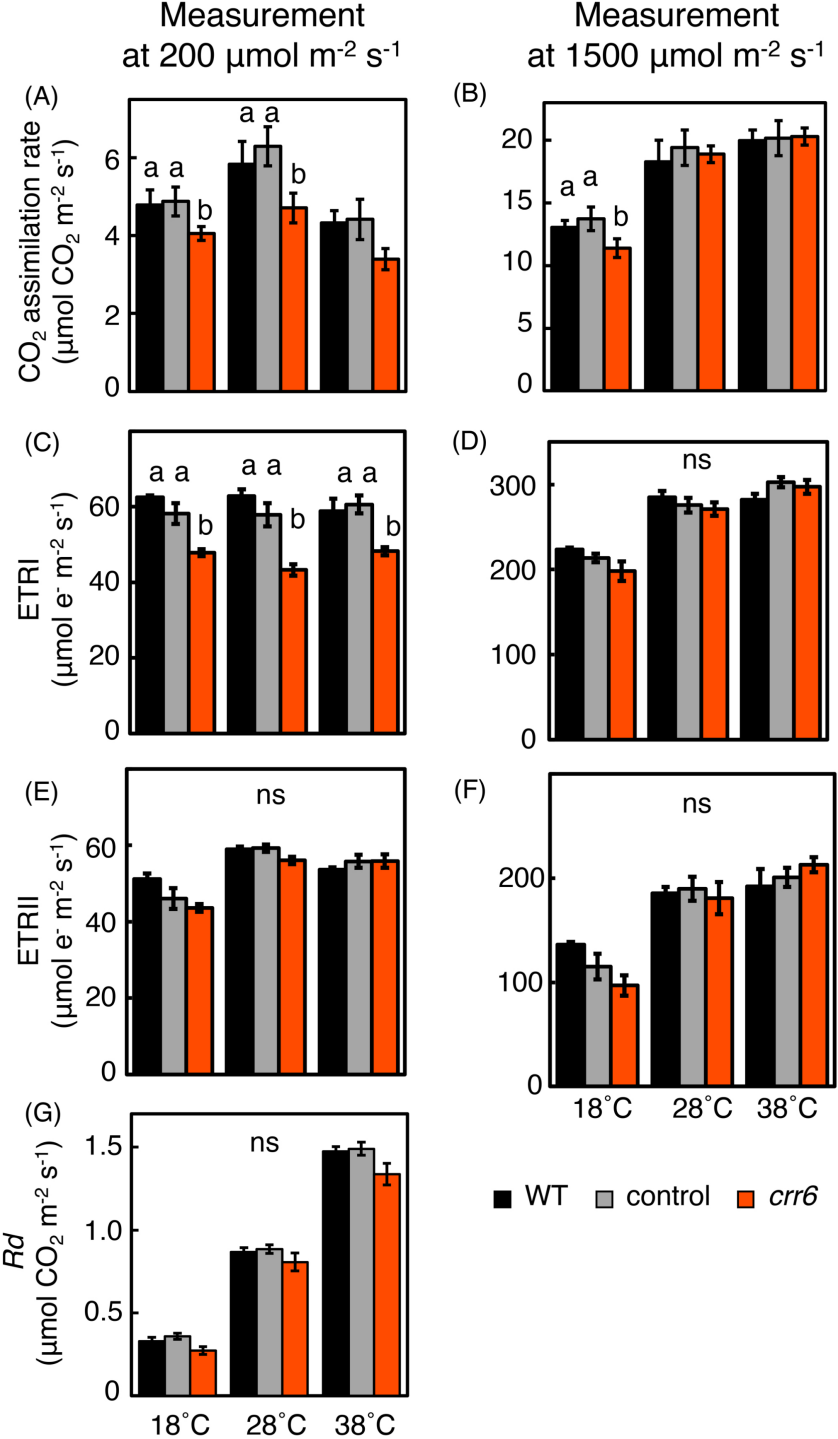
Comparison of photosynthetic parameters under low and high irradiance at different temperatures. Values of (A, B) net CO_2_ assimilation rate, (C, D) electron transport rate through PSI [ETR(I)], (E, F) electron transport rate through PSII [ETR(II)], and (G) dark respiration rate (*R*_e_) at each temperature were extracted from the light‐response curves shown in Figures 3 and 4 and compared between low light (200 μmol m^−2^ s^−1^) and high light (1500 μmol m^−2^ s^−1^). Values represent means ± SE (*n* = 3–5). Significant differences among genotypes were evaluated using Tukey–Kramer tests (*p* < 0.05). Different letters indicate significant differences among WT, control, and *crr6* plants according to Tukey–Kramer multiple comparison tests (*p* < 0.05).

Light‐response curves of photosynthetic parameters were measured at 18°C, 28°C, and 38°C (Figures 2, 3, 4). The *crr6* mutant exhibited a significantly lower CO_2_ assimilation rate under relatively weak light, although the affected light range depended on temperature. At 28°C and 38°C, differences were observed between 200 and 800 μmol m^−2^ s^−1^, whereas at 18°C, reductions occurred across nearly the entire light range (Figure 2A–C; Figure 4A,B). On the other hand, NDH deficiency had no significant effect on stomatal conductance (*g*_s_), intercellular CO_2_ concentration (*C*_*i*_), or dark respiration rate (*Rd*) across temperatures and light intensities (Figure 2D–I; Figure 4G). Accompanied with a decrease in CO_2_ assimilation rate, ETRI was significantly lower in the *crr6* mutant below 1000 μmol m^−2^ s^−1^ across all temperatures (Figure 3A–C; Figure 4C,D). Consistent with this reduction in PSI electron transport, Y(NA) was elevated in the *crr6* mutant under sub‐saturating light. Y(NA) increased below 1000 μmol m^−2^ s^−1^ at 18°C and 28°C and under 500 μmol m^−2^ s^−1^ at 38°C, while Y(ND) did not differ significantly between lines under these conditions (Figure 3D–I).

ETRII was largely similar among lines except at moderate light intensities (500–750 μmol m^−2^ s^−1^) at 18°C while ETRI was strongly correlated with CO_2_ assimilation rate (Figure 3J–L; Figure 4E,F). However, the PQ pool was more reduced in the *crr6* mutant as indicated by higher 1 − qL values at 200–500 μmol m^−2^ s^−1^ at 18°C and 28°C (Figure 3M–O). NPQ did not differ significantly between lines under these conditions (Figure 3P–R).

The ratio ETRI/ETRII, commonly used as an indicator of CET activity, was significantly lower in the *crr6* mutant under low light at 28°C and 38°C, suggesting reduced CET capacity. There was no difference, but ETRI/ETRII tended to be higher than WT in the *crr6* mutant at 18°C.

Together, these results indicate that NDH deficiency leads to limited electron flow at PSI caused by high PSI acceptor‐side limitation. Along with this, enhanced reduction of the PQ pool under relatively low irradiance, particularly, thereby contributes to reduced CO_2_ assimilation. This phenomenon was more remarkable as measurement temperature decreased.

### 3.5 NDH Deficiency Exacerbates Photosynthetic Decline Under Fluctuating Light

**FIGURE 5:**
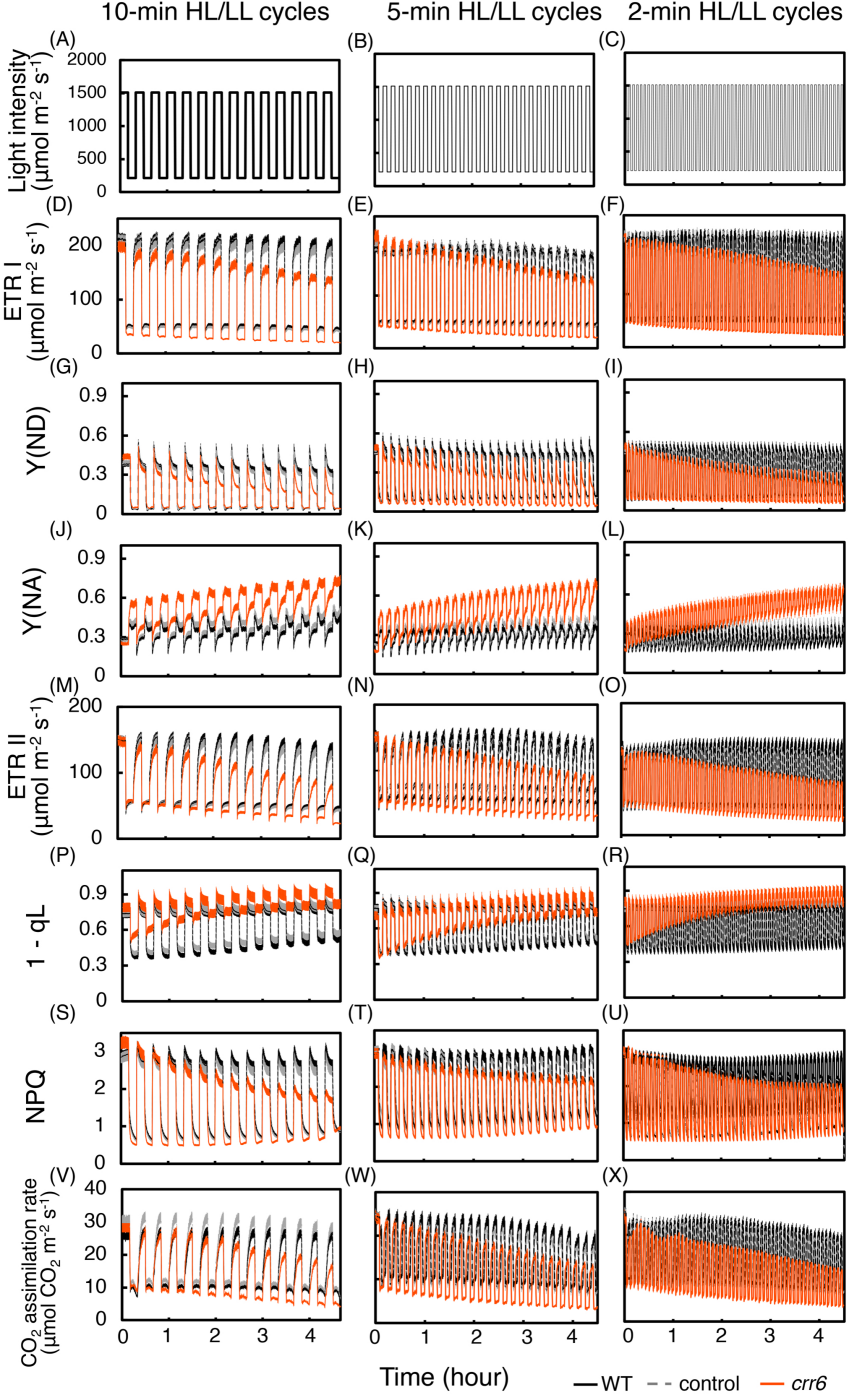
Photosynthetic responses to fluctuating light in WT and *crr6* plants. Photosynthetic parameters were measured at an ambient CO_2_ concentration of 400 μmol mol^−1^ under three fluctuating light regimes consisting of repeated cycles between high light (1500 μmol m^−2^ s^−1^) and low light (200 μmol m^−2^ s^−1^) for 4.5 h in field‐grown plants. Light fluctuations were applied at intervals of 10 min (A, D, G, J, M, P, S, V), 5 min (B, E, H, K, N, Q, T, W), or 2 min (C, F, I, L, O, R, U, X). Parameters shown are (A–C) measurement light conditions, (D–F) electron transport rate through PSI [ETR(I)], (G–I) donor‐side limitation of PSI [Y(ND)], (J–L) acceptor‐side limitation of PSI [Y(NA)], (M–O) electron transport rate through PSII [ETR(II)], (P–R) fraction of closed PSII centers (1 − qL), (S–U) non‐photochemical quenching (NPQ), and (V–X) net CO_2_ assimilation rate. Values represent means ± SE (*n* = 3–5).

Photosynthetic responses were examined under three fluctuating‐light regimes (10‐min HL/LL cycles, 5‐min HL/LL cycles, 2‐min HL/LL cycles; Figure 5A–C; Figure S2A–C). Initial photosynthetic parameters were consistent with the steady‐state light‐response measurements. Under fluctuating light, PSI redox dynamics differed among lines. ETRI gradually declined in both genotypes; however, the decline was markedly greater in the *crr6* mutant during both high‐ and low‐light phases (Figure 5D–F; Figure S2D–F; Figure S3A–C; Figure S4A–C; Figure S5A–C). In WT plants, Y(ND) and Y(NA) remained relatively stable throughout the treatment. In contrast, the *crr6* mutant showed a decrease in Y(ND) at 1500 μmol m^−2^ s^−1^ and ultimately fell to less than half of the WT levels (Figure 5G–I; Figure S2G–I; Figure S3D–F; Figure S4D–F; Figure S5D–F). Simultaneously, Y(NA) progressively increased, reaching approximately 40% higher levels than WT under both light intensities (Figure 5J–L; Figure S2J–L; Figure S3G–I; Figure S4G–I; Figure S5G–I). This means PSI was gradually more reduced in the *crr6* mutant.

Accompanied by the ETRI decrease, ETRII also gradually declined more noticeably in the *crr6* mutant (Figure 5M–O; Figure S2M–O; Figure S3J–L; Figure S4J–L; Figure S5J–L). Furthermore, the PQ pool was more reduced in the mutant at both light intensities, as the mutant showed high 1‐qL, similar to over‐reduced PSI (Figure 5P–R; Figure S2P–R; Figure S3M–O; Figure S4M–O; Figure S5M–O). Consistent with PQ over‐reduction, NPQ induction was also suppressed, particularly during the high‐light phases in the mutant (Figure 5S–U; Figure S2S–U; Figure S3P–R; Figure S4P–R; Figure S5P–R). As a result, CO_2_ assimilation rate gradually declined more greatly in the *crr6* mutant; consequently, the final values of these parameters were substantially lower in the mutant than in WT plants (Figure 5V–X; Figure S2V–X; Figure S3S–U; Figure S4S–U; Figure S5S–U). These findings indicate that NDH‐dependent CET is essential for maintaining PSI redox balance and sustaining electron transport under fluctuating light, independent of fluctuation frequency.

### 3.6 NDH‐Dependent CET Protects PSI From Photoinhibition Under Fluctuating Light

**FIGURE 6:**
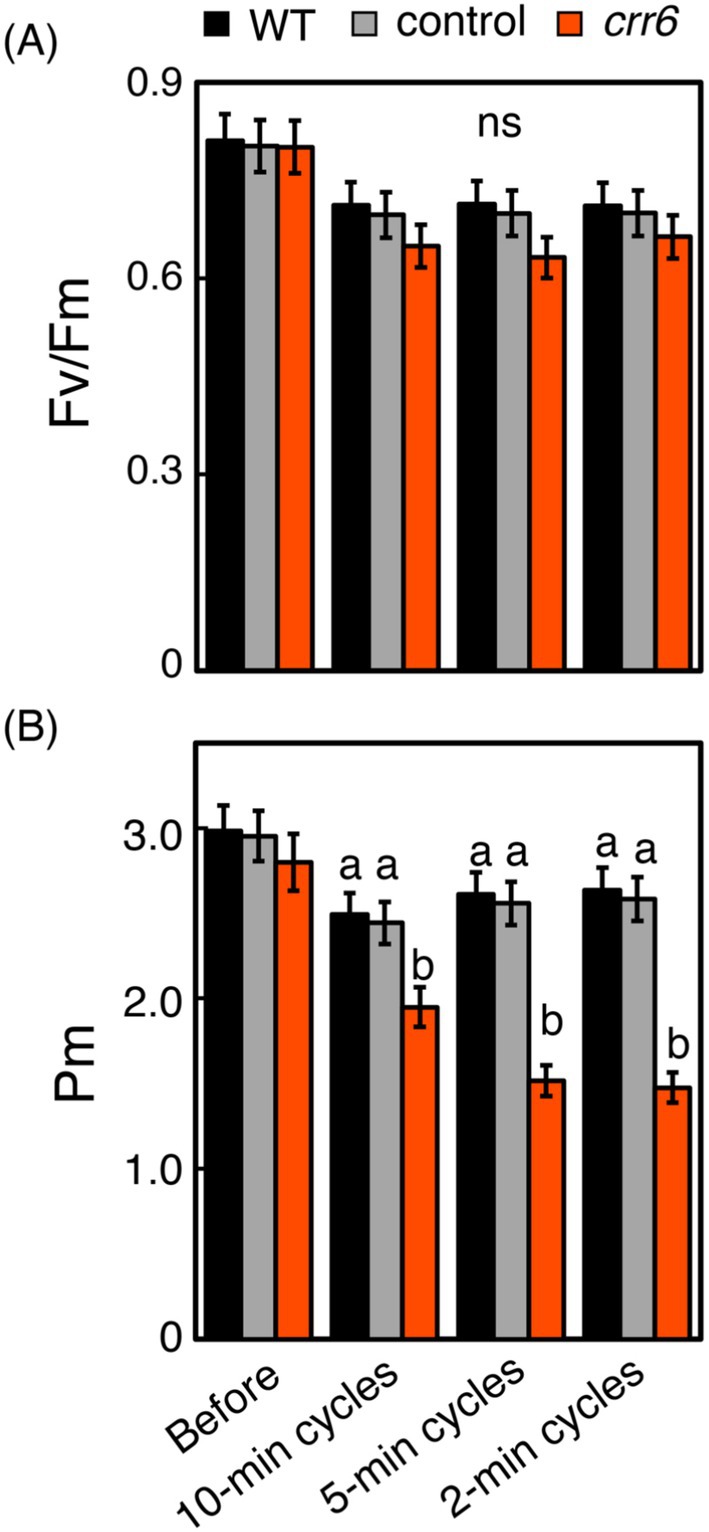
Photoinhibition of PSI and PSII following fluctuating light treatment in WT and *crr6* plants. Photoinhibition parameters were measured following the fluctuating light treatments shown in Figure 5. Leaves were exposed to repeated cycles of high light (1500 μmol m^−2^ s^−1^) and low light (200 μmol m^−2^ s^−1^) for 4.5 h at intervals of 10, 5, or 2 min. After the treatment, leaves were dark‐incubated for 30 min prior to measurements. (A) Maximum quantum yield of PSII (F_v_/F_m_). (B) Maximum oxidizable P700 signal of PSI (P_m_). Values represent means ± SE (*n* = 3–5). Different letters indicate significant differences among genotypes according to Tukey–Kramer multiple comparison tests (*p* < 0.05).

To assess photoinhibition, maximum PSII quantum yield (Fv/Fm) and maximum P700 signal (Pm) were measured before and after 4.5 h of fluctuating light treatment (Figure 6). Before treatment, both parameters were similar among genotypes. After fluctuating light exposure, both Fv/Fm and Pm decreased in all lines. This indicates fluctuating light induced photoinhibition of both PSI and PSII. However, Pm declined significantly in the *crr6* mutant, while Fv/Fm declined to a similar extent in all lines, indicating that PSI photoinhibition was more pronounced in the *crr6* mutant. Moreover, PSI photodamage became more severe as fluctuation frequency increased, demonstrating that NDH‐dependent CET plays a critical role in protecting PSI under rapidly changing light environments.

## 4 Discussion

In natural field environments, plants are exposed to continuous fluctuations in irradiance and temperature that can constrain photosynthetic performance. However, the physiological significance of NDH‐dependent cyclic electron transport in plants grown under such realistic conditions has remained unclear. In this study, we investigated the physiological relevance of NDH‐dependent CET by combining growth analyses under naturally fluctuating cultivation conditions with detailed physiological measurements conducted under controlled experimental environments. The mutant exhibited reduced CO_2_ assimilation during fluctuating light treatments (Figure 5V–X), which was associated with significant decreases in biomass accumulation and grain yield (Figure 1C–E). Physiological measurements further revealed that NDH deficiency impaired PSI electron transport and carbon fixation, particularly under sub‐saturating irradiance and low temperature (Figures 2, 3, 4). These findings indicate that NDH‐dependent CET plays an important role in sustaining photosynthetic efficiency and plant productivity when crops experience dynamically changing environmental conditions in the field. There are two limitations of the present study. One is that detailed photosynthetic measurements were performed under controlled experimental conditions rather than directly under cultivation conditions. Therefore, the mechanistic relationship between reduced photosynthetic performance and decreased productivity remains partly inferential. Nevertheless, the consistency between environmentally dependent impairments in PSI electron transport and the growth reduction observed under naturally fluctuating cultivation conditions supports the physiological importance of NDH‐dependent CET under realistic environmental conditions. The other is that the present study used only one mutant lacking the NDH complex. Therefore, it is desirable to use complementation lines and additional NDH‐deficient mutants to strengthen the understanding of NDH‐dependent CET as we showed in the present study.

### 4.1 NDH‐Dependent CET Sustains PSI Electron Transport and Carbon Assimilation Under Weak Light and Low Temperature Conditions Frequently Encountered in the Field

A central finding of this study is that NDH deficiency primarily impaired PSI electron transport and CO_2_ assimilation under sub‐saturating irradiance at a wide range of temperatures. At moderate (28°C) and high (38°C) measurement temperatures, the reduction in CO_2_ assimilation in the *crr6* mutant was confined mainly to the low‐to‐intermediate light range (Figure 2A–C), whereas at low temperature (18°C), the impairment extended across nearly the entire light response curve. This temperature‐dependent expansion of the phenotype strongly suggests that NDH‐CET is important for maintaining photosynthetic capacity when biochemical carbon fixation capacity is restricted in field‐grown rice.

Importantly, this reduction in assimilation was not associated with changes in stomatal conductance, respiration rate, or the abundance of major photosynthetic components such as Rubisco, chlorophyll, cytochrome *b*_6_/*f*, or leaf nitrogen content (Figure 2D–I; Figure 4G; Table 1). Instead, the suppression of CO_2_ assimilation closely paralleled the reduction in ETRI, indicating that NDH deficiency primarily limits the regeneration phase of the Calvin–Benson cycle through an altered energy balance rather than stomatal CO_2_ diffusional or structural limitations. Because RuBP regeneration frequently limits photosynthesis under weak irradiance (Hikosaka et al. 2006), the loss of NDH‐mediated *pmf* formation likely reduces ATP supply relative to NADPH, thereby constraining carbon fixation.

This interpretation is consistent with previous reports demonstrating that NDH‐dependent CET contributes to *pmf* formation by coupling Fd oxidation to proton translocation across the thylakoid membrane (Strand et al. 2017) and supports photosynthesis under weak light in rice (Yamori et al. 2015). At low temperatures, the activity of Calvin–Benson cycle enzymes becomes kinetically restricted, which is one of the main factors promoting over‐reduction of stromal electron acceptors and increasing susceptibility of PSI to photoinhibition (Sonoike 2025). NDH‐CET likely alleviates this imbalance by enhancing photosynthetic control at the cytochrome *b*_6_/*f* complex and stabilizing PSI activity. Indeed, earlier work demonstrated that NDH‐deficient rice shows decreased PSI activity and CO_2_ assimilation under chilling conditions (Yamori et al. 2011), a phenomenon further supported by the broader light‐range impairment observed here at 18°C. In addition to this, intercellular CO_2_ concentration of the *crr6* mutant tended to be higher than the WT at 18°C along with a decrease of the CO_2_ assimilation rate. This observation supports the idea that the reduced assimilation rate was primarily caused by biochemical limitations, such as insufficient ATP supply associated with impaired NDH‐dependent CET, particularly under low‐temperature conditions.

Field environments are characterized by prolonged exposure to sub‐saturating irradiance due to canopy shading, cloud movement, and diurnal solar geometry (Pearcy 1990). Moreover, in temperate rice‐growing regions, crops are frequently exposed to moderate or low temperatures throughout much of the growing season. Consequently, NDH‐dependent CET is likely to play a particularly important ecological role under environmental scenarios where weak irradiance and low temperature co‐occur. Because the duration of strong irradiance exceeding 1000 μmol m^−2^ s^−1^ is typically limited during a day, cumulative carbon gain in the field is largely determined by photosynthetic performance under sub‐saturating light. The reduced biomass accumulation and grain yield observed in *crr6* plants (Figure 1C–E) can therefore be interpreted as an integrated outcome of chronically reduced photosynthesis during these environmentally prevalent conditions. This interpretation is consistent with the well‐established relationship between leaf photosynthetic capacity and plant productivity (Evans 2013; Yamori, Kondo, et al. 2016).

It should also be noted that alternative energy dissipation pathways may partially compensate for NDH deficiency. Previous studies suggested that enhanced photorespiration or activation of other cyclic or alternative electron transport routes can contribute to maintaining PSI redox balance when NDH function is impaired (Peterson et al. 2016; Chen et al. 2023). The relatively small difference in the CET activity index (ETRI/ETRII) between genotypes at low temperature observed here may support the possibility that additional regulatory mechanisms operate under such conditions. Further investigation will be required to clarify the detailed metabolic adjustments underlying the reduced assimilation in NDH‐deficient plants.

The physiological roles of CET pathways remain under active discussion, and the relative contributions of each pathway to maintaining photosynthesis and protecting PSI are not yet fully understood. Our results suggest that NDH‐dependent CET contributes to both photosynthetic regulation and PSI protection in rice grown under naturally fluctuating cultivation conditions.

### 4.2 NDH‐Dependent CET Stabilizes PSI Redox Balance and Prevents Cumulative PSI Photoinhibition Under Fluctuating Light

Under simulated fluctuating light regimes, NDH‐deficient plants exhibited progressive declines in ETRI, ETRII, and CO_2_ assimilation (Figure 5D–F,M–O,V–X), accompanied by stepwise increases in acceptor‐side limitation of PSI, as indicated by elevated Y(NA), and decreases in donor‐side limitation Y(ND). These responses resulted in a reduction in maximum PSII quantum yield (Fv/Fm) and maximum P700 signal (Pm) after fluctuating light treatment, indicating photoinhibition of both PSI and PSII (Figure 6). The *crr6* mutant exhibited significant PSI photoinhibition, while the extent of PSII photoinhibition was not different among lines.

Such vulnerability of PSI under fluctuating irradiance has been reported previously in NDH‐deficient rice and Arabidopsis (Yamori, Makino, and Shikanai 2016; Kono et al. 2017; Zhou et al. 2023). Mechanistically, CET pathways appear to play complementary roles during dynamic light transitions. PGR5‐dependent CET and NDH‐dependent CET contribute to maintaining PSI oxidation through donor‐side regulation and acceptor‐side regulation. Donor‐side regulation rapidly induces ΔpH‐dependent photosynthetic control, thereby restricting electron flow toward PSI through proton‐gradient formation (Munekage et al. 2004; Suorsa et al. 2012; Takeuchi et al. 2025). In contrast, acceptor‐side regulation alleviates PSI over‐reduction by transferring electrons from reduced Fd back to the PQ pool via NDH‐dependent CET (Zhou et al. 2022). Together, these mechanisms help prevent excessive reduction of the PSI acceptor side under fluctuating light conditions. Based on our light‐response analyses and previous studies investigating the physiological role of NDH, this acceptor‐side regulatory function of NDH‐dependent CET appears to become particularly important during low‐light phases, when PSI acceptor‐side limitation is enhanced because of delayed activation of stromal metabolism (Yamori et al. 2015; Zhou et al. 2023).

Interestingly, although the interval of light fluctuation did not markedly affect the magnitude of assimilation decline, PSI photoinhibition tended to become more severe as fluctuation frequency increased (Figure 6). These findings suggest that cumulative carbon gain and PSI structural stability respond differently to temporal light dynamics. In natural field environments where irradiance can change on timescales of seconds, the ability of NDH‐CET to facilitate rapid redox recovery of PSI acceptors may represent an important adaptive mechanism.

One important question is whether PSI photoinhibition accumulates in the natural environment. When we measured Pm before fluctuating light irradiation, the Pm value was not different between WT and *crr6* mutant suggesting that PSI photodamage was relatively limited during cultivation. This may indicate that light fluctuations under greenhouse conditions were less severe than those imposed in our artificial fluctuating‐light experiments. On the other hand, PSI photoinhibition is thought to begin with damage to the functional F_A_ and F_B_ iron–sulfur clusters (Sonoike 2025), which may not immediately affect P700 measurements (Tiwari et al. 2016). Therefore, early‐stage PSI damage could potentially accumulate without large detectable changes in Pm. Taken together, these observations suggest that repeated exposure to fluctuating light, weak irradiance, and low temperature may cumulatively impair PSI electron transport and CO_2_ assimilation in the *crr6* mutant under naturally fluctuating cultivation conditions, although the direct contribution of PSI photoinhibition remains difficult to quantify in the present study.

### 4.3 Field Environments Integrate Multiple Environmental Drivers That Amplify NDH Phenotypes

Natural terrestrial environments impose complex combinations of environmental constraints on photosynthesis (Yamori 2016). Consistent with this ecological framework, NDH‐deficient mutants exhibited substantial reductions in growth and yield under field cultivation despite showing relatively modest differences in biochemical leaf traits. These results indicate that the phenotypic impact of NDH deficiency likely reflects the cumulative effects of recurrent exposure to weak light, low temperatures, fluctuating irradiance, and dynamic spectral environments. Under such realistic growth conditions, NDH‐dependent cyclic electron transport appears to function as a key stabilizing mechanism that maintains long‐term photosynthetic performance and crop productivity. As pointed out by Yamori, Makino, and Shikanai (2016); Yamori, Kondo, et al. (2016), the contribution of NDH‐dependent CET to photosynthetic regulation and plant growth appears to be greater in rice than in Arabidopsis, in which NDH‐deficient mutants show only subtle phenotypes. One possible explanation is that rice has a greater demand for *pmf* formation and a more limited capacity of alternative electron transport pathways to compensate for the loss of NDH‐dependent CET.

## 5 Conclusions

This study demonstrates that NDH‐dependent cyclic electron transport contributes to sustaining photosynthetic efficiency and productivity in rice cultivated under naturally fluctuating environmental conditions. NDH deficiency led to impaired PSI electron transport, reduced CO_2_ assimilation, and consequent decreases in biomass and grain yield. These effects became particularly evident under environmentally relevant conditions such as weak irradiance, low temperature, and fluctuating light. Our findings indicate that NDH‐CET contributes to maintaining photosynthetic robustness in dynamic terrestrial environments and represents an important regulatory component supporting crop performance under realistic growth conditions.

## Author Contributions

W.Y. conceived the study and designed the experiments. H.K. and W.Y. performed the experiments and H.K. analyzed the data with contributions from W.Y. H.K., and H.K. and W.Y. wrote the manuscript and approved the final version.

## Funding

This work was supported by: JSPS KAKENHI grant 21H02171 (W.Y.); JST ALCA‐Next (Grant Number JPMJAN25D2 to W.Y.).

## Conflicts of Interest

The authors declare no conflicts of interest.

## Supporting information


**Figure S1:** Characterization of the NDH‐deficient *crr6* plants.
(A) Immunoblot analysis of photosynthetic proteins. CRR6 is a stromal protein required for accumulation of NDH subcomplex A, whereas NDHK is a subunit of this subcomplex. Cytochrome f (Cyt *f*) is a component of the cytochrome b_6_/f complex. Total leaf proteins were separated by SDS PAGE and detected using the indicated antibodies. Proteins were loaded on an equal leaf are basis. (B) Thylakoid membrane protein complexes isolated from wild‐type (WT), control, and crr6 plants. Thylakoid membranes were solubilized with 1.0%, 1.5%, or 2.0% (w/v) dodecyl maltoside and separated by blue native‐PAGE. Gels were stained with Coomassie Brilliant Blue. Equal amounts of chlorophyll were loaded per lane. (C) NDH activity monitored by chlorophyll fluorescence analysis. A representative fluorescence trace from WT plants is shown. Arrows indicate the timing of measuring light (ML) and actinic light (AL; 200 μmol m^−2^ s^−1^). The transient increase in fluorescence following AL cessation (boxed region) was used as an indicator of NDH activity. Enlarged traces from WT, control, and *crr6* plants are shown below.
**Figure S2:** Individual plots for WT, control, and *crr6* plants corresponding to Figure 5.
Photosynthetic parameters under fluctuating light regimes in WT, control, and *crr6* plants. Light fluctuations were applied at intervals of 10 min (A, D, G, J, M, P, S, V), 5 min (B, E, H, K, N, Q, T, W), or 2 min (C, F, I, L, O, R, U, X). Parameters shown are (A–C) incident light conditions, (D–F) electron transport rate through PSI [ETR(I)], (G–I) donor‐side limitation of PSI [Y(ND)], (J–L) acceptor‐side limitation of PSI [Y(NA)], (M–O) electron transport rate through PSII [ETR(II)], (P R) fraction of closed PSII centers (1 − qL), (S–U) non‐photochemical quenching (NPQ), and (V–X) net CO_2_ assimilation rate. Values represent means ± SE (*n* = 3–5).
**Figure S3:** Photosynthetic parameters during 10‐min high‐light/low‐light cycles corresponding to Figure 5.
Photosynthetic parameters under fluctuating light regimes consisting of alternating high light and low light at 10‐min intervals. Parameters shown are (A–C) electron transport rate through PSI [ETR(I)], (D–F) donor‐side limitation of PSI [Y(ND)], (G–I) acceptor‐side limitation of PSI [Y(NA)], (J–L) electron transport rate through PSII [ETR(II)], (M–O) fraction of closed PSII centers (1 − qL), (P–R) non‐photochemical quenching (NPQ), and (S–U) net CO_2_ assimilation rate. Values represent means ± SE (*n* = 3–5).
**Figure S4:** Photosynthetic parameters during 5‐min high‐light/low‐light cycles corresponding to Figure 5.
Photosynthetic parameters under fluctuating light regimes consisting of alternating high light and low light at 5‐min intervals. Parameters shown are (A–C) electron transport rate through PSI [ETR(I)], (D–F) donor‐side limitation of PSI [Y(ND)], (G–I) acceptor‐side limitation of PSI [Y(NA)], (J–L) electron transport rate through PSII [ETR(II)], (M–O) fraction of closed PSII centers (1 − qL), (P–R) non‐photochemical quenching (NPQ), and (S–U) net CO_2_ assimilation rate. Values represent means ± SE (*n* = 3–5).
**Figure S5:** Photosynthetic parameters during 2‐min high‐light/low‐light cycles corresponding to Figure 5.
Photosynthetic parameters under fluctuating light regimes consisting of alternating high light and low light at 2‐min intervals. Parameters shown are (A–C) electron transport rate through PSI [ETR(I)], (D–F) donor‐side limitation of PSI [Y(ND)], (G–I) acceptor‐side limitation of PSI [Y(NA)], (J–L) electron transport rate through PSII [ETR(II)], (M–O) fraction of closed PSII centers (1 − qL), (P–R) non‐photochemical quenching (NPQ), and (S–U) net CO_2_ assimilation rate. Values represent means ± SE (*n* = 3–5).

## Data Availability

The data that support the findings of this study are available on request from the corresponding author. The data are not publicly available due to privacy or ethical restrictions.
