# Supplementary material for: Cyclic Electron Transport via the NDH Complex Sustains Photosynthesis and Productivity Under Fluctuating and Sub‐Optimal Environments
Source: Physiol Plant. 2026 Jul 6;178(4):e71004. doi: 10.1111/ppl.71004 (PMC13337337; doi:10.1111/ppl.71004)
Supplement: Supplementary file 1 — Figure S1: Characterization of the NDH‐deficient crr6 plants. (A) Immunoblot analysis of photosynthetic proteins. CRR6 is a stromal protein required for accumulation of NDH subcomplex A, whereas NDHK is a subunit of this subcomplex. Cytochrome f (Cyt f) is a component of the cytochrome b6/f complex. Total leaf proteins were separated by SDS PAGE and detected using the indicated antibodies. Proteins were loaded on an equal leaf are basis. (B) Thylakoid membrane protein complexes isolated from wild‐type (WT), control, and crr6 plants. Thylakoid membranes were solubilized with 1.0%, 1.5%, or 2.0% (w/v) dodecyl maltoside and separated by blue native‐PAGE. Gels were stained with Coomassie Brilliant Blue. Equal amounts of chlorophyll were loaded per lane. (C) NDH activity monitored by chlorophyll fluorescence analysis. A representative fluorescence trace from WT plants is shown. Arrows indicate the timing of measuring light (ML) and actinic light (AL; 200 μmol m−2 s−1). The transient increase in fluorescence following AL cessation (boxed region) was used as an indicator of NDH activity. Enlarged traces from WT, control, and crr6 plants are shown below. Figure S2: Individual plots for WT, control, and crr6 plants corresponding to Figure 5. Photosynthetic parameters under fluctuating light regimes in WT, control, and crr6 plants. Light fluctuations were applied at intervals of 10 min (A, D, G, J, M, P, S, V), 5 min (B, E, H, K, N, Q, T, W), or 2 min (C, F, I, L, O, R, U, X). Parameters shown are (A–C) incident light conditions, (D–F) electron transport rate through PSI [ETR(I)], (G–I) donor‐side limitation of PSI [Y(ND)], (J–L) acceptor‐side limitation of PSI [Y(NA)], (M–O) electron transport rate through PSII [ETR(II)], (P R) fraction of closed PSII centers (1 − qL), (S–U) non‐photochemical quenching (NPQ), and (V–X) net CO2 assimilation rate. Values represent means ± SE (n = 3–5). Figure S3: Photosynthetic parameters during 10‐min high‐light/low‐light cycles corresponding [file PPL-178-e71004-s001.pdf]

Title

Cyclic electron transport via the NDH complex sustains photosynthesis and productivity under fluctuating and sub-optimal environments

Running title

NDH sustains photosynthesis under dynamic and sub-optimal environments

Names of all authors

Hiromasa Kodama & Wataru Yamori*

Names and addresses of the institutions where the work was carried out

Graduate School of Agricultural and Life Sciences, The University of Tokyo, Nishitokyo, Tokyo, Japan

*Corresponding author: Wataru Yamori, Institute for Sustainable Agro-ecosystem Services, Graduate School of Agricultural and Life Sciences, The University of Tokyo, Tokyo 188-0002, Japan. E-mail: yamori@g.ecc.u-tokyo.ac.jp, ORCID: 0000-0001-7215-4736

Email address for each author

Hiromasa Kodama (salamander-nut444@g.ecc.u-tokyo.ac.jp)

Wataru Yamori: (yamori@g.ecc.u-tokyo.ac.jp)

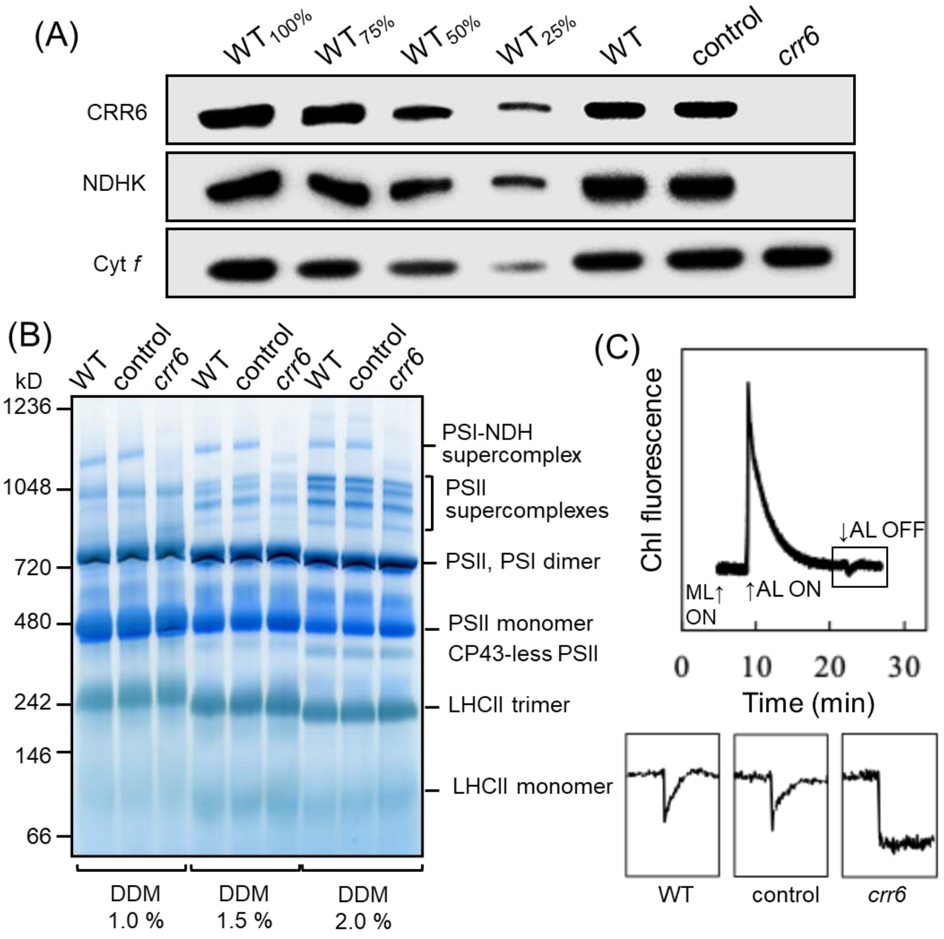

Figure S1. Characterization of the NDH-deficient *crr6* plants

(A) Immunoblot analysis of photosynthetic proteins. CRR6 is a stromal protein required for accumulation of NDH subcomplex A, whereas NDHK is a subunit of this subcomplex. Cytochrome *f* (Cyt *f*) is a component of the cytochrome b_6/f complex. Total leaf proteins were separated by SDS-PAGE and detected using the indicated antibodies. Proteins were loaded on an equal leaf are basis. (B) Thylakoid membrane protein complexes isolated from wild-type (WT), control, and crr6 plants. Thylakoid membranes were solubilized with 1.0%, 1.5%, or 2.0% (w/v) dodecyl maltoside and separated by blue native-PAGE. Gels were stained with Coomassie Brilliant Blue. Equal amounts of chlorophyll were loaded per lane. (C) NDH activity monitored by chlorophyll fluorescence analysis. A representative fluorescence trace from WT plants is shown. Arrows indicate the timing of measuring light (ML) and actinic light (AL; 200 μmol m^{-2} s^{-1}). The transient increase in fluorescence following AL cessation (boxed region) was used as an indicator of NDH activity. Enlarged traces from WT, control, and *crr6* plants are shown below.

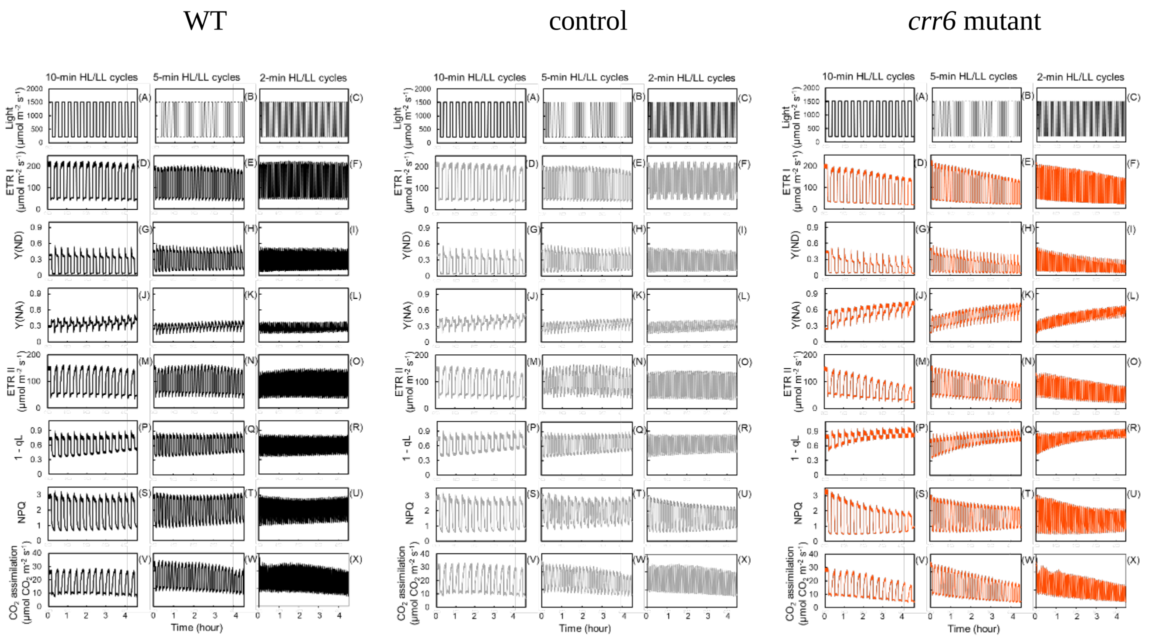

Figure S2. Individual plots for WT, control, and *crr6* plants corresponding to Figure 5. Photosynthetic parameters under fluctuating light regimes in WT, control, and *crr6* plants. Light fluctuations were applied at intervals of 10 min (A, D, G, J, M, P, S, V), 5 min (B, E, H, K, N, Q, T, W), or 2 min (C, F, I, L, O, R, U, X). Parameters shown are (A–C) incident light conditions, (D–F) electron transport rate through PSI [ETR(I)], (G–I) donor-side limitation of PSI [Y(ND)], (J–L) acceptor-side limitation of PSI [Y(NA)], (M–O) electron transport rate through PSII [ETR(II)], (P–R) fraction of closed PSII centers (1 − qL), (S–U) non-photochemical quenching (NPQ), and (V–X) net CO_2 assimilation rate. Values represent means ± SE (n = 3–5).

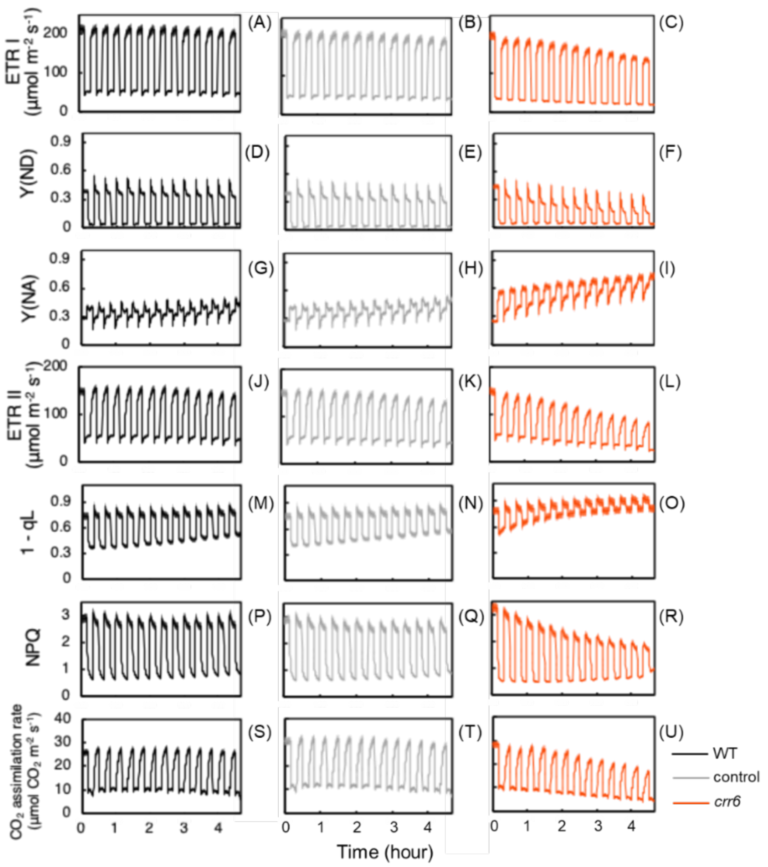

Figure S3. Photosynthetic parameters during 10-min high-light/low-light cycles corresponding to Figure 5.

Photosynthetic parameters under fluctuating light regimes consisting of alternating high light and low light at 10-min intervals. Parameters shown are (A–C) electron transport rate through PSI [ETR(I)], (D–F) donor-side limitation of PSI [Y(ND)], (G–I) acceptor-side limitation of PSI [Y(NA)], (J–L) electron transport rate through PSII [ETR(II)], (M–O) fraction of closed PSII centers (1 − qL), (P–R) non-photochemical quenching (NPQ), and (S–U) net CO_2 assimilation rate. Values represent means ± SE (n = 3–5).

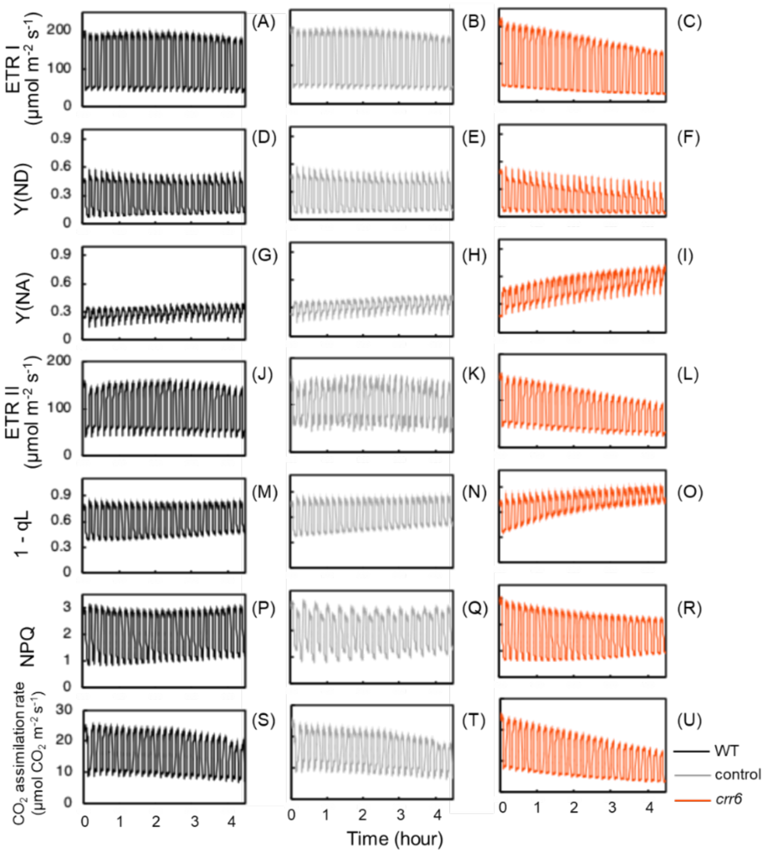

Figure S4. Photosynthetic parameters during 5-min high-light/low-light cycles corresponding to Figure 5.

Photosynthetic parameters under fluctuating light regimes consisting of alternating high light and low light at 5-min intervals. Parameters shown are (A–C) electron transport rate through PSI [ETR(I)], (D–F) donor-side limitation of PSI [Y(ND)], (G–I) acceptor-side limitation of PSI [Y(NA)], (J–L) electron transport rate through PSII [ETR(II)], (M–O) fraction of closed PSII centers (1 − qL), (P–R) non-photochemical quenching (NPQ), and (S–U) net CO_2 assimilation rate. Values represent means ± SE (n = 3–5).

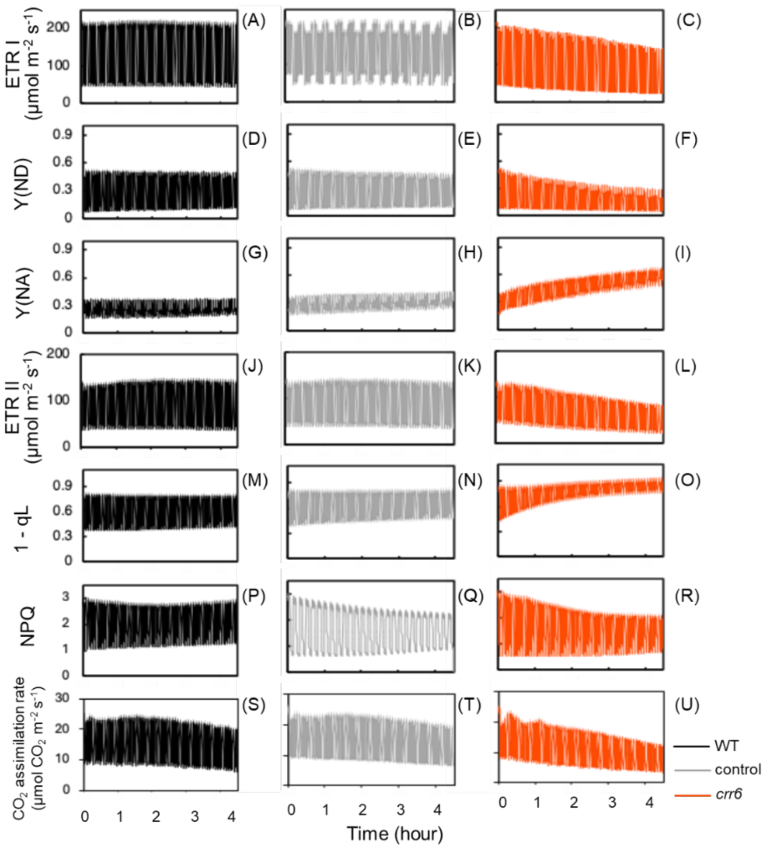

Figure S5. Photosynthetic parameters during 2-min high-light/low-light cycles corresponding to Figure 5.

Photosynthetic parameters under fluctuating light regimes consisting of alternating high light and low light at 2-min intervals. Parameters shown are (A–C) electron transport rate through PSI [ETR(I)], (D–F) donor-side limitation of PSI [Y(ND)], (G–I) acceptor-side limitation of PSI [Y(NA)], (J–L) electron transport rate through PSII [ETR(II)], (M–O) fraction of closed PSII centers (1 − qL), (P–R) non-photochemical quenching (NPQ), and (S–U) net CO_2 assimilation rate. Values represent means ± SE (n = 3–5).
